# A model of the interrelationship between research ethics and research integrity

**DOI:** 10.1080/17482631.2023.2295151

**Published:** 2023-12-21

**Authors:** Abdulghani Muthanna, Youmen Chaaban, Saba Qadhi

**Affiliations:** a Department of Teacher Education, Norwegian University of Science and Technology, Trondheim, Norway; b Educational Research Center, Qatar University, Doha, Qatar; c Core Curriculum Program, Qatar University, Doha, Qatar

**Keywords:** Research ethics, research integrity, research misconduct, plagiarism, fabrication

## Abstract

**Purpose:** The purpose of this article is to explore the interrelationship between research ethics and research integrity with a focus on the primary forms of research misconduct, including plagiarism, fabrication, and falsification. It also details the main factors for their occurrence, and the possible ways for mitigating their use among scholars.

**Methods:** The method employed a detailed examination of the main ethical dilemmas, as delineated in literature, as well as the factors leading to these ethical breaches and the strategies to mitigate them. Further, the teaching experiences of the primary author are reflected in the development of the model.

**Results:** The results of this article are represented in a model illustrating the interrelationship between research ethics and research integrity. Further, a significant aspect of our article is the identification of novel forms of research misconduct concerning the use of irrelevant or forced citations or references.

**Conclusion:** In conclusion, the article highlights the substantial positive effects that adherence to research ethics and integrity have on the academic well-being of scholars.

## 1. Introduction

There is a strong interrelationship between research ethics and research integrity. While research ethics are principles and regulations for researchers to follow in conducting scientific research, research integrity is the practice of these codes. The violation of the research ethics then engenders the presence of research misconduct that reflects, at least, some lack of research integrity.

This article aims to point out the interrelationship between the two concepts: research ethics and research integrity. Meanwhile, it also aims to discuss the primary forms of research misconduct such as plagiarism, fabrication, and falsification. In each of these forms, we have also tried to detail the main factors for its occurrence, and the possible ways for decreasing or fighting against it. Further, our article attempts to bring the attention of researchers to the existence of a new form of research misconduct that relates to the use of irrelevant citations or references that researchers need to cite so that their works get published in specific journals.

Our article also provides a model that shows the interrelationship between research ethics and research integrity. By understanding this simple model, it becomes easier for scholars to avoid falling into the trap of research misconduct, and thus strengthening their research integrity. Additionally, the article mentions the critical positive impacts of following and practicing research ethics and research integrity on the well-being of scholars. Below is a brief discussion of the research integrity concept.

## 2. Research integrity

Research integrity primarily concerns researchers’ actions and behaviours following the rules and regulations governing their disciplines. Regardless of whether humans or animals are recruited as participants in a research endeavour, researchers must conform to a set of agreed-upon practices that communicate the *integrity* of the published work. Research integrity gains importance within a research community by allowing its members to trust and build on previous work while also developing confidence in their scholarly outputs within society (Bouter, 2023). Trust in the research system is thus a result of research integrity.

Given the complexity of research integrity and its variation as a function of discipline, context, and culture, a universal definition may be hard to find. It may be best to understand research integrity concerning researchers’ actions and behaviours that conform to the highest standards of professionalism and rigour when conducting research in ethically robust ways (Hiney, 2015). The use of meaning-loaded words, such as professionalism, rigour, and robustness, to describe integrity creates space for researchers, policymakers, and the public to develop principles of integrity, as is evident in diverse documents (e.g., the Cape Town Statement of Fairness, Equity, and diversity in Research (Horn et al., 2023), the European Code of Conduct for Research Integrity (ALLEA, 2023), and the Global Research Council’s (2022) Statement of Principles and Practices for Research Ethics, Integrity, and Culture in the Context of Rapid-Results Research).

These statements, which employ positive language, share common objectives related to research integrity, yet diverge in the specific principles they emphasize. It is worth noting that all of these statements use positive language to emphasize desirable actions and behaviours that researchers should prioritize when upholding research integrity. However, their differences are significant, likely influenced by various factors such as national variations, recent developments (e.g., the acknowledgement of indigenous knowledge), and lack of universal consensus.

Further, the dynamism of research integrity has been influenced by recent technological advancements, such as the proliferation of Natural Language Programs (NLPs) (e.g., ChatGPT), and their capability to create scholarly work. These tools have divided the research community. While some have embraced their widespread availability, and urged for the necessity of regulating matters related to integrity, transparency, and authorship, others have chosen to completely ban their use (Hosseini et al., 2023). For instance, Hosseini et al. recommend that these tools should not be listed as authors or acknowledged, citing their lack of free will and accountability. However, they can still be cited within the text and included in the references. The impact of these tools on research integrity remains a subject of ongoing debate.

A parallel movement has been to document violations of research integrity using prohibitive language, sometimes within the same document (e.g., ALLEA, 2023). There have been numerous cases of research misconduct, as violations are commonly termed, that have renounced *good* researcher practices. The most widely cited misconduct includes fabrication, falsification, and plagiarism, yet may further include multiple other violations that blemish researcher profiles and the institutions they represent. These questionable research practices (QRPs) are more subtle and prevalent than fabrication, falsification and plagiarism (FFP). Some examples of QRPs, as documented in ALLEA (2023), include:

- Allowing funders to interfere with the research process or report
- Misusing seniority by encouraging violations or artificially advancing one’s career by using the work of others
- Hampering other researchers’ work
- Using automated tools in writing (e.g., NLPs)
- Dissecting research results across multiple publications that do not advance knowledge about the research problem and analyse the same results in the same way
- Misrepresenting authorship by claiming undeserved author contributions or denying deserved contributions
- Publishing the same work in a different language without acknowledgement
- Participating in cartels of researchers who review and accept each other’s work

Whether these and other QRPs qualify as research misconduct are not straightforward and may depend on several considerations (DuBois et al., 2013). For instance, the seriousness of the behaviours and the degree of liability of the researcher should be considered (DuBois et al., 2013; Fanelli & Tregenza, 2009). Because this misconduct is not readily visible in published manuscripts, it may be difficult to detect them explicitly in published work. When undetected for some time, they may “become a part of a self-deceptive narrative that will make the next transgression easier” (Hiney, 2015, p. 5). Accordingly, such violations are more prevalent than FFP and more difficult to contain (O’Boyle & Gotz, 2022). For example, there is the practice of data exploration, known as “data mining,” where researchers retrospectively seek statistically significant relationships and present them as the study’s primary objective, without clearly disclosing their analysis as an exploratory analysis (Bornmann, 2013; Nurunnabi & Hossain, 2019). Furthermore, their variation across disciplines and cultures leads to a more significant variation in regulatory responses. Research documenting the frequency of research misconduct provides different numbers but generally agrees that its prevalence among researchers deserves careful attention (Bouter, 2023; Fanelli & Tregenza, 2009; Reisig et al., 2020).

To counter questionable research practices, the community involving all stakeholders should examine the core reasons leading to problematic behaviours. According to DuBois et al. (2013), narcissistic thinking and traits play a role in research misconduct. Other researchers remove some of the blame from individual researchers and point towards the university systems, including cultures and structures inherent to a highly competitive environment (Bouter, 2023; O’Boyle & Gotz, 2022). Researchers may feel pressure from promotion structures that generally rely on research publications in high-impact journals or find funding applications in low supply and high demand greatly competitive (Bouter, 2023; Hiney, 2015). With caution, O’Boyle and Gotz (2022) propose the analogy of comparing QRPs with criminal justice violations, illustrating the means, motive, and opportunity that drive researchers into wrongdoing. While the means are QRPs, the motive relates to higher chances of publication success, and the opportunity resides in researchers *getting away with it* without detection. Yet again, they contend that “this is a system problem, not a person problem” (p. 274). Pressure on researchers to publish, garner funds, and secure citations can lead many, intentionally or unintentionally, to engage in QRPs (Bouter, 2023).

As most research activity is connected to either financial incentives or career advancement, there needs to be strict regulatory systems that guide researchers in maintaining trust and punitive procedures that hold them accountable for misconduct. Again, this discussion should also consider the distinction between research misconduct and QRPs. In regards to the latter, O’Boyle and Gotz (2022) classify actionable steps into three categories, namely ”(1) reducing the motivation to engage in QRPs, (2) increasing transparency in reporting, and (3) embracing methodological rigor” (p.274). Bouter (2023) calls for scholarly journals and funding agencies to adopt open science practices and for researchers to engage in preregistering studies ahead of data collection and analysis. However, it is unclear how preregistering studies will help maintain research integrity, despite ensuring that there exists a complete data set of all studies conducted on a particular subject. This shows how research integrity is also an issue for journals and publishers, which should also publish negative results, though they do not qualify to the same degree as positive results. Before research misconduct occurs, Hiney (2015) discusses the importance of training programmes that aim at educating researchers about proper research conduct, thus increasing conformity and enhancing knowledge about the consequences of FFP. Equally important are the characteristics of the environment in which researchers work; the level of competition, the availability of funds, and the degree of organizational justice play an essential role in leveraging good research practices (Bouter, 2023). In short, responsibility for research integrity should be monitored by the institution where researchers are employed, regulated by national systems, and scrutinized using international agreements and guidelines.

## 3. Research ethics and research misconduct

Research ethics is a set of principles, laws, and regulations, along with ethical considerations (we use *“codes”* to include all concepts) that researchers must follow for conducting scientific research (Sharma, 2015). The heart of research ethics is *trust, honesty, consciousness, and professional commitment* (European Science Foundation, 2011; European Science Foundation Policy Debriefing, 2000; Insuring Integrity in Irish Research, 2010; Steneck, 2007; Superior Council of Scientific Investigations SCSI, 2021). While ethical standards are a matter of international concern (Resnik et al., 2015), it is crucial to recognize that these standards can vary between cultures. Furthermore, these variations in ethical standards can extend to differences from one institution to another within the same cultural context (Bloch, 2008). This highlights the need for researchers to be mindful of these cultural and institutional differences when addressing issues of research integrity to ensure their work adheres to the specific ethical standards relevant to their particular context.

It is positive that many international organizations, such as the European Science Foundation, Council of Science Editors, and Committee on Publication Ethics, to name a few, have worked to issue international policies regulating the ethics of conducting scientific research and raising a global research community. Although international and national research misconduct policies help raise awareness of research misconduct (Resnik et al., 2015), research ethics are still violated. The violation of research ethics engenders research misconduct, ranging from fabrication and/or falsification of data to plagiarism (Patrzek et al., 2014). We detail these primary research misconducts below.

### 3.1 Plagiarism

Plagiarism is an old phenomenon with labels such as literary theft (Park, 2003), academic crime, intellectual dishonesty, or failing, to name a few (Hu & Lei, 2015). Misconducts showing plagiarism vary; however, the main ones are associated with data ownership, collection tools and procedures, and dissemination (Steneck, 2007). In other words, plagiarism could be intentional, wherein an author intentionally takes the works/words of other scholars without crediting them (Patrzek et al., 2014; Perry, 2010). Another is unintentional, wherein an author commits plagiarism due to a lack of proper referencing guidelines or poor language skills (Perry, 2010). It could also be self-plagiarism by repeating the same ideas or publishing a work in different journals at different periods (Roig, 2010). Roig (2010) also reported other common forms of self-plagiarism: augmented publication and segmented publication (salami publication). Salami plagiarism is the process of publishing two or more papers based on the findings of one study which essentially recycle and present the same results and analyses in a repetitive manner, instead of advancing knowledge on a particular issue or subject (Abraham, 2000, as cited in; Smolčić, 2013). In such cases, publishing multiple papers based on a single study does not contribute any novel insights or value to existing literature, and the repetition of results and analytical approaches raises ethical concerns regarding the transparency and integrity of the research process.

Another plagiarism form is patchwriting, also known as mosaic writing, wherein an author takes some text parts from different sources and tailors them together, making it hard for software tools to detect plagiarism (Roig, 2010; Smolčić & Bilić-Zulle, 2013). Although re-phrasing the ideas of others without acknowledgement is the most common form of plagiarism, committing any of these forms is unethical. There are many multidimensional actors for the happening of such unethical research misconduct. Lack of academic norms (insufficient academic research exposure) and limited communicative competence in English are, on the other hand, reported to be the critical factors behind plagiarism (Howard, 1999; Hu & Lei, 2015). Online publications also ease the process of copying and pasting (Park, 2003), forming another factor of plagiarism. Further, competing for funds, craving for fame, and/or intending to hurt colleagues are possible factors for plagiarism (Organization for Economic Cooperation and Development: Global Science Forum, n.d..). Meanwhile, the lack of correspondence with other researchers/colleagues, awareness of proper scientific research standards, and high pressure from supervisors or publishers for more significant results (Organization for Economic Cooperation and Development: Global Science Forum, n.d..) are also possible factors.

To fight against plagiarism, researchers must consider such misconduct an issue of worldwide concern for all communities, who must continuously warn their members against it. In other words, it is a shared responsibility of all engaged in science (Muthanna, 2016). Further, researchers can use the “prevention” strategy, which highlights the full understanding of the causes of the phenomenon and provides immediate remedies, and the seemingly harsh, yet in some cases necessary the “deterrence/enforcement” that targets the exclusion of plagiarists from the scientific community as an example for deterring other committers of such scientific misconduct (Organization for Economic Cooperation and Development: Global Science Forum, n.d..). These two strategies provide many steps as remedies for the plagiarism problem. For example, using computer-assisted tools, unification/promotion of international standards for publication, rewarding quality rather than quantity, presence of open and frank discussions on the problem of plagiarism, the incorporation of clear instructions on the issue in the curricula and training both instructors and students on such instructions are all possible solutions for reducing the spread of such misconduct in the world of science (Organization for Economic Cooperation and Development: Global Science Forum, n.d.., p. 13).

Moreover, authors in general, and editors and reviewers in particular, are advised to join the Committee on Publication Ethics (COPE) established in 1997 to advise editors and reviewers and fight against research misconduct. Several (audio and written) individual cases of misconduct can be learned from or used by instructors as examples of defaming one’s personality. Further, it is recommended to read the published code of conduct that provides clear guidelines for editors about best practices on editorial and peer-review processes (Committee on Publication Ethics, 2011).

### 3.2. Fabrication

Fabrication is a form of research misconduct in which researchers intentionally falsify and invent research data that were either never collected or distorted in some way to support a specific hypothesis or conclusion (Fanelli & Tregenza, 2009; Hiney, 2015). Put simply, it uses false or fake data, fake participants, and/or fake consent forms.

In investigating multiple forms of research misconduct using survey data, Reisig et al. (2020) operationalized data fabrication along several dimensions, including fabricating data for desired results, writing a more competitive research grant, providing additional statistical validity, making results from a pilot study more attractive, and inventing data that was never collected. However, the authors found research fabrication to be the least perceived research misconduct among other forms. While Fanelli and Tregenza (2009) claims that fabrication remains “the more problematic category” (p. 1), it should be emphasized that it is equally serious as other forms of misconduct.

With the recent proliferation of NLP systems, Hosseini et al. (2023) argue that such tools have made it easier for researchers to fabricate transcripts of interviews or answers to open-ended questions. They also discuss the uses of NLPs in generating literature reviews and synthesizing thematic ideas. While the editors of *Accountability in Research* encourage authors to use NLPs, they emphasize the need to disclose all instances of NLP assistance during the research process. The intersection of ethics, research integrity, and policy related to NLP systems will remain a *hot topic* among researchers and editors and continue to garner necessary attention on contending with research fabrication using NLP systems.

### 3.3 Falsification

Similar to fabrication, falsification is a practice that strikes at the core of scientific inquiry. Falsification, a deceptive practice in the scholarly domain, involves deliberate manipulations, fabrications, or distortions of research data or results orchestrated to misrepresent the veritable essence of scientific inquiry (Martyn, 2003; Timothy et al., 2001). The complex issue demonstrates a spectrum of misleading strategies, encompassing subtle alterations of data and blatant fabrication (Fanelli & Tregenza, 2009). Within the realm of data falsification exist intricate approaches that contain tactics such as data manipulation, referred to as “cooking,” which involves assigning excessive importance to specific data elements. Another practice of equal importance involves deliberately publishing only supportive results, avoiding the publication of incongruent outcomes. Moreover, data smoothing, which involves the unwarranted removal of anomalous data points, contributes to a distorted portrayal of the truth.

The ethical dilemma of falsification in research is multifaceted and extends beyond its narrow conceptualization. This instance violates the core principles that form the basis of the scientific community’s endeavour to acquire knowledge. The systematic study and meta-analysis conducted by Fanelli and Tregenza (2009) shed light on the prevalence of this phenomenon, revealing that a significant number of researchers had been involved in fabricating or falsifying data at least once over their professional trajectories. The issue at hand is not limited to a certain field but instead spans other study areas, underscoring the significance of tackling this problem from a holistic perspective.

The complex and varied expressions of deception highlight the diverse character of this unethical behaviour. According to Earp and Trafimow (2015), using strategies such as data manipulation and selective data analysis demonstrates deliberate attempts to obtain results that support predetermined hypotheses, compromising the objectivity required in scientific inquiry. The intentional omission of some findings not only distorts the storytelling aspect of the research procedure, but also undermines the shared repository of knowledge, thereby diminishing the confidence that both the scientific community and the general public have in study conclusions.

One significant issue from falsification is the possibility of consequential ripple effects. Modifying even a solitary data point can potentially disseminate misinterpretations, influencing subsequent research endeavours, policy formulations, and public discussions. Kang (2020) highlights the negative outcomes of this type of misconduct, which include impeding scientific advancement and wasting important resources that could have been allocated to reputable research endeavours. Zietman (2013) emphasizes the importance of adopting a complete strategy to tackle research misconduct, including the implementation of strict rules, the establishment of effective oversight mechanisms, and a dedication to cultivating a research environment that upholds integrity.

Combating against falsification necessitates the collaborative endeavours of researchers, institutions, and the broader scientific community. Implementing measures to guarantee openness, replicability, and the widespread distribution of comprehensive datasets is crucial in addressing this matter. The promotion of collaborative initiatives aimed at educating researchers about the ethical implications of falsification and its consequences has the potential to instigate a transformative culture change towards enhanced integrity. In essence, the reinforcement of research ethics is not solely a duty but rather a shared obligation aimed at safeguarding the integrity and advancement of scientific endeavours.

## 4. New forms of research misconduct: irrelevant or forced citation

Unsurprisingly, researchers have entered the endless competitive arena of publications (for job promotions, incentives, and/or job security) (e.g., Feng et al., 2013; Lee & Lee, 2013). Also, it is not surprising that the competition has become confined to the SCI and/or SSCI journals in most if not all, cases. However, it is surprising that the higher the journal impact factor is, the better it is thought to be. It stands to note that such an impact factor could be deceptive as citations could also be negative. Further, there is a significant increase in the networking process (known as local or international collaboration) among authors, which is positive unless the focus is on citing one another, even though many other papers are more relevant to the researched topic(s). Put differently; there is a tendency towards citing one another when there are at least tens of other more pertinent papers that Zavrsnik et al. (2016) termed as *“sleeping beauties.”* More surprisingly, some journals demand citing their published works without considering that they might be less relevant or irrelevant to the new submissions. These dangerous issues deserve attention: purposive but less relevant citations or forced citations are, therefore, new forms of research misconduct that we all should combat. In short, it is critical that reviewing submissions should strictly focus on checking the relevance and the topic-in-depth of the cited references while also comparing these references to the sleeping beauties in the field.

## 5. The interrelationship between research ethics and research integrity

**Figure 1.**
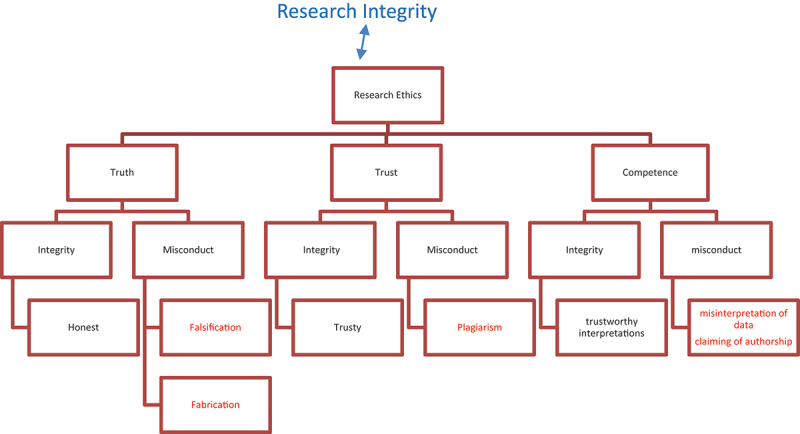
A model of the interrelationship between research ethics and research integrity.

Considering the above discussions, research ethics are the codes scholars need to follow for conducting scientific research. If scholars behave according to the given codes, it is a practical reflection/application of research integrity mainly concerned with behaviour. If scholars’ behaviour does not adhere to the precepts stipulated by research ethics, there is a violation of research integrity; research integrity not only encompasses research ethics as an umbrella value but also reflects how these research ethics are realized in reality. Drawing upon the classification of research misconduct by Kuroki (2018), the first author has also developed the following model, shown in Figure 1, based on teaching “research integrity” for graduate and post graduate candidates for five years. Figure shows the interrelationship between research integrity and research ethics in connection to ”*truth, trust, and competence.”*

The above model shows how research ethics relate to realizing research integrity when practiced with integrity. Meanwhile, it also shows how violating research ethics reflects a lack of integrity. It also shows how research ethics and research integrity relate to each other so that they cannot be separated.

As the primary purpose of conducting scientific research is to report the “truth” (reality), researchers who do not fabricate data (e.g., use of fake/false data, addition of data, or participants’ fake consent forms) or falsify research (e.g., inappropriate manipulation of data such as changing or omission of data, results or hypotheses), they are then considered to be honest and integrative, reflecting “truth” as it occurs in reality. However, if researchers commit either fabrication or falsification, they are fabricating or falsifying the truth.

By connecting the research misconduct of “plagiarism” (e.g., intentional or unintentional plagiarism in the form of “salami: augmented or segmented; mosaic (copy and paste); or cheating in exams) with ‘trust’ as the heart of research ethics (European Science Foundation, 2011), there is then a violation of ‘trust’ the societies have given researchers (Kuroki, 2018). We trust that researchers do not use the words, thoughts, or ideas of others without accrediting them properly; otherwise, they not only destroy ‘trust’ but also commit serious research misconduct related to ‘plagiarism.’ On the other hand, when researchers accredit their colleagues” words, thoughts, and ideas, it reflects their being “trusty,” a value that reflects the application of research integrity.

Finally, we believe that the “competence” of researchers is one classification of research ethics. It relates to using multiple skills focusing on “the ability to interpret” the collected data correctly and sincerely. Researchers must be well-trained not only on how to collect data but also on how to interpret it. This competence leads to providing both “true and trusty” (trustworthy) findings; if not, it is then a misinterpretation of data or a violation of both “truth and trust” in the big picture. Suppose researchers are not competent and do not reflect their competence in research. In that case, it is then possible that they might co-author research without any right, leading to a lack of both research ethics and research integrity.

To conclude, while research ethics relates to a set of codes researchers need to follow masterfully, research integrity is the practical application/reflection of these ethics in reality. Research integrity relates to researchers’ behaviour and behaviour while putting research ethics into practice. In other words, research ethics and research integrity complement each other, and we cannot separate them in words or actions.

## 6. Key positive impacts behind achieving research ethics and research integrity on scholars’ health and well-being

Following research ethics and behaving morally (applying research integrity) in conducting scientific research has many positive impacts on the well-being of scholars. Among many, the scholars reflect that they are honest and trustworthy, motivating the global community to continue their trust and feel secure to get recruited as study participants when requested. This trust also continues to thrive among the scholars’ students, who would see their scholars/instructors as competent role models.

By following and practicing both research ethics and integrity in conducting research, scholars show that the qualifications they have obtained are warranted and that they can guide their students well on how to conduct scientific research.

## 7. Conclusion

In summary, the concept of research integrity stands as a critical pillar of scientific endeavour and holds great significance within the scientific community. Integrity pertains to researchers’ adherence to the established norms and standards of their specific fields. The underlying premise of this argument is predicated on the notion that scholarly investigations ought to adhere to principles of integrity, comprehensiveness, and independence from ideological, economic, or political biases. Trust plays a crucial role in upholding research integrity, enabling scientists to leverage prior research and establish credibility for their scholarly contributions within the broader societal context.

The concept of research integrity is multidimensional and can exhibit variations across different fields, contexts, and cultures. Although a precise and universally accepted definition may be challenging to establish, the concept can be most comprehensively grasped as the adherence of researchers to elevated standards of professionalism and ethical rigour in their activities and behaviours. Numerous international documents and statements, including but not limited to the Cape Town Statement of Fairness, Equity, and Diversity in Research, the European Code of Conduct for Research Integrity, and the Global Research Council’s Statement of Principles and Practices for Research Ethics and Integrity, underscore the significance of research integrity and offer fundamental principles to guide scholarly pursuits.

Plagiarism, fabrication, and falsification are prevalent forms of research misconduct that pose equal and significant danger to the integrity of scholarly inquiry. Plagiarism encompasses the act of utilizing someone else’s work without appropriate acknowledgement. It can arise from a multitude of circumstances, such as disparities in cultural norms and the weight of academic expectations. In order to address the issue of plagiarism, it is imperative to foster consciousness, establish global benchmarks, prioritize excellence above quantity, and offer explicit instructions for researchers.

Fabrication, which refers to the deliberate creation or alteration of research data, remains a prominent issue. The emergence of advanced natural language processing (NLP) techniques has introduced fresh complexities in this domain. The proper utilization of Natural Language Processing (NLP) necessitates the inclusion of disclosure and ethical deliberations. Falsification, which encompasses the deliberate modification and distortion of research data, is an unethical practice that undermines the integrity of scientific impartiality. The phenomenon in question can manifest itself in diverse manners, including but not limited to data manipulation, the deliberate selection of certain information for reporting, and the deletion of relevant details without proper justification. Similar to fabrication, the requirement to address falsification necessitates the presence of transparency, replicability, and the widespread distribution of comprehensive datasets.

The significance of upholding the pertinence and calibre of references in scientific endeavours is underscored by the advent of novel instances of research misconduct, including extraneous or coerced citations, which also reveals how research integrity is an issue for journals and publishers. Our model shows that the fundamental connection between research ethics and research integrity has significant importance. Research ethics encompass a collection of principles and guidelines that govern the conduct of research. In contrast, research integrity pertains to how these principles and procedures are implemented in practical research endeavours. Engaging in research practices that contravene ethical guidelines signifies a deficiency in upholding the principles of research integrity, whereas adhering to such guidelines fosters trust, competence, and the advancement of knowledge in research.

The attainment of research ethics and integrity has a beneficial effect on the mental well-being of scholars, enabling them to engage in research activities with a sense of moral rectitude. Additionally, it promotes the development of trust among colleagues and students, serves as an exemplar for ethical conduct, and ensures the establishment of academics’ standing within the academic community. In essence, the interconnection between research integrity and ethics is of utmost importance in facilitating the advancement of scientific knowledge. Adhering to these principles maintains research integrity, cultivates confidence, and adds to the overall welfare of researchers and society.
